# Association of Genotyping of *Bacillus cereus* with Clinical Features of Post-Traumatic Endophthalmitis

**DOI:** 10.1371/journal.pone.0147878

**Published:** 2016-02-17

**Authors:** Meng Hong, Qian Wang, Zhide Tang, Youpei Wang, Yunfeng Gu, Yongliang Lou, Meiqin Zheng

**Affiliations:** 1 Department of Clinical Laboratory Medicine, the Affiliated Eye Hospital of Wenzhou Medical University, Wenzhou, 325027, China; 2 Key Laboratory of Laboratory Medicine, Ministry of Education, Zhejiang Provincial Key Laboratory of Medical Genetics, Wenzhou Medical University, Wenzhou, Zhejiang, 325035, China; Fudan University, CHINA

## Abstract

*Bacillus cereus* is the second most frequent cause of post-traumatic bacterial endophthalmitis. Although genotyping of *B*. *cereus* associated with gastrointestinal infections has been reported, little is known about the *B*. *cereus* clinical isolates associated with post-traumatic endophthalmitis. This is largely due to the limited number of clinical strains available isolated from infected tissues of patients with post-traumatic endophthalmitis. In this study, we report successful isolation of twenty-four *B*. *cereus* strains from individual patients with different disease severity of post-traumatic endophthalmitis. Phylogenetic analysis showed that all strains could be categorized into three genotypes (GTI, GTII and GTIII) and the clinical score showed significant differences among these groups. We then further performed genotyping using the *vrrA* gene, and evaluated possible correlation of genotype with the clinical features of *B*. *cereus*–caused post-traumatic endophthalmitis, and with the prognosis of infection by conducting follow-up with patients for up to 2 months. We found that the disease of onset and final vision acuity were significantly different among the three groups. These results suggested that the *vrrA* gene may play a significant role in the pathogenesis of endophthalmitis, and genotyping of *B*. *cereus* has the potential for predicting clinical manifestation and prognosis of endophthalmitis. To the best of our knowledge, this is the first report of isolation of large numbers of clinical isolates of *B*. *cereus* from patients with endophthalmitis. This work sets the foundation for future investigation of the pathogenesis endophthalmitis caused by *B*. *cereus* infection.

## Introduction

*Bacillus cereus* is an endospore-forming, gram-positive rod that is ubiquitous in the environment under either aerobic or anaerobic conditions. *B*. *cereus* is commonly reported as a pathogenic bacterium for food poisoning[1]. Although intraocular infections caused by *B*. *cereus* are rare, it is the second most frequent cause of post-traumatic bacterial endophthalmitis, approximately occurring in 2–7% of all penetrating wounds to the eye, with most cases related to trauma or surgery[2, 3].Post-traumatic endophthalmitis has a devastating complication, in which patients develop endophthalmitis or even panophthalmitis within 48h after infection[4].The vision outcomes are generally poor, with 75–91% of patients experiencing a loss of light perception, evisceration or enucleation[5]. Although some patients recovered final vision acuity better than 2/100, or even complete recovery of visual acuity after treatment[6], recovery of useful vision (better than 2/100) from *B*. *cereus* endophthalmitis is not common. Because of its capability to cause blindness quickly and aggressively with endophthalmitis, *B*. *cereus* is regarded as an important ocular pathogen which must been taken seriously.

Most of the genome sequencing and genotyping studies have been reported on the environmental *B*. *cereus* strains or clinical isolates associated with gastrointestinal infections[1,7–9]. On the other hand, limited studies have been reported on genotyping and genomic information about the *B*. *cereus* clinical isolates associated with post-traumatic endophthalmitis, largely due to the limited number of clinical strains available.

Several genes, such as *groEL* and *sodA*, have been reported to be utilized for detection and differentiation of close related *Bacillus species*, or among *B*. *cereus* strains[10, 11]. However, since the sequences of these genes are evolutionary conserved, they are not very effective for genotyping of close-related *B*. *cereus* isolates. In this regard, analysis of repetitive-element sequence polymorphism, in particular, the tandem repeats in the variable region of the *vrrA* gene, has been suggested as a means of differentiating between different strains of *B*. *cereus*[12–14].The purpose of this study is to 1) isolate *B*. *cereus* from clinical samples of post-traumatic endophthalmitis patients with different severity of diseases and prognosis; 2) conduct *vrrA* gene-based genotyping on *B*. *cereus* isolates; and 3) examine a possible relationship between the genotypes and clinical representation or prognosis of *B*. *cereus* endophthalmitis.

## Materials and Methods

### Patients and clinical observations

Patient samples were collected in either Eye Hospital of Wenzhou Medical University or Zhongshan Ophthalmic Center, Sun Yat-sen University between January 2010 and December 2012. Ethical approvals for sample collection were obtained from the Ethics Committees in both Eye Hospital of Wenzhou Medical University and Zhongshan Ophthalmic Center, Sun Yat-sen University. Conduction of genotyping of *Bacillus cereus* using these pre-collected samples was initiated on May, 2015, which was approved by the Ethics Committee of Eye Hospital of Wenzhou Medical University(KYK[2015]34).

For this retrospective study, the patient inclusion criteria are as follows: infective endophthalmitis caused by penetrating wounds; Gram stains for vitreous biopsy specimen and foreign bodies all positive; *B*. *cereus* that grows on the culture of vitreous humor and aqueous humor; without any history of ocular injury and systemic disease; without any other infective disease. Thus, twenty-four patients (22 males and 2 females) with a mean age of 44.5±17.1 years (range: 6–82 years) were included in this retrospective study. The follow-up time was 21.1±8.8 months (range: 12–38 months) (**Table 1**). The resource of penetrating trauma was as follows: iron (n = 14), pebbles (n = 7), branch (n = 1), electric wire (n = 1) and firecracker (n = 1). Additionally, all patients provided written informed consent before enrollment.

**Table 1:** Patients’ Information.

Variables	Values
Demographic data	
Age (years)	44.5±17.1 (6–82)
Sex (M/F)	22/2
Occupation	
Worker	16
Farmer	6
Student	2
Baseline characteristics	
Laterality (left/right)	13/11
Character of foreign body (n)	
Iron	14
Pebbles	7
Branch	1
Electricity wire	1
Firecracker	1
Size of wound(mm^2^)	4.2±2.2 (1–8)
Onset time (h)	32.2±39.2 (2–168)
Follow-up time (m)	21.1±8.8 (12–38)
Treatment (n)	
Enucleation	6
Vitrectomy	17
Removal of the foreign body	1
Complication of eye (n)	
Complicated cataract	12
Atrophy of eyeball	3
Previous surgical procedures (n)	0

Most patients (n = 21) developed rapid and aggressive ocular pain, redness, thick yellowish discharge, and notably reduced visual acuity. The typical clinical findings were severe swelling of the eyelids, conjunctival chemosis, conjunctival congestion, corneal edema, and hypopyon. All patients were performed slit lamp examination, indirect ophthalmoscopic examination and B-scan before and after surgery operation. In order to describe the clinical manifestation, we used the clinical grading scale reported by Kim JY et al[15].

### Bacterial strains

A total of twenty-four *B*. *cereus* strains were isolated from clinical infective endophthalmitis in Eye Hospital of Wenzhou Medical University and Zhongshan Opthalmic Center, Sun Yet-sen University. All strains were isolated from sterile collection of anterior chamber fluid or vitreous body, and identified by API 20E and API 50CHB strip identification. To determine the specificity of the PCR primers for *vrrA* amplication, *B*. *cereus* ATCC14579, *B*. *thuringiensis* CTCC22945, *B*. *subtilis* ATCC9372, *Staphylococcus aureus* ATCC25923, *Staphylococcus epidermidis* ATCC12228, *Strep tococcus* ATCC12344, *Pseudomonas aeruginosa* ATCC14953, *Escherichia coli* ATCC25922, *Proteus vulgaris* CMCC49072, and *Enterobacter aerogenes* ATCC13048 were used in parallel. The *B*. *cereus*, *B*. *thuringiensis* and *B*. *subtilis* standard strains were from Wenzhou Center for Disease Control, Wenzhou, China. The standard strains of the other seven non-*B*. *cereus* groups were obtained from Zhejiang Provincial Center of Biological Experiment Teaching, Zhejiang, China. All strains were grown overnight at 37°C on blood agar medium under an atmosphere of 5% CO_2_, and were used for DNA extraction.

### PCR amplification of *vrrA* gene

Template DNA was extracted from isolated colonies with the TaKaRa MiniBEST universal genomic DNA extraction kit (TaKaRa) according to the manufacturer’s instruction. We designed a pair of primers to PCR the virable region of *vrrA* according to the public *vrrA* sequence (*vrrA*-F: 5'-AATGTATGAATCAAACGAAA-3', Reference GenBank accession, *vrrA*-R 5'-AGTGGATAGAAAACAAAGGA-3', and Reference NC_00472.1). The PCR reaction mixture contained 2μl of template DNA, 10 mM Tris-HCl (pH 8.3), 50 mM KCl, 1.5mM MgCl2, 0.2 mM of each deoxynucleoside triphosphate, 0.2μM of each primer, and 1.25U of Taq DNA polymerase (TaKaRa). The total volume was 50μl. Ultrapure water (without DNA) was used as negative control. The anticipated length of PCR products were 432bp. The PCR protocols were described as follows: 95°C for 5min; 94°C for 60s, 56°C for 45s, 72°C for 60s for 32cycles, and 72°C for 10min. The process was repeated twice per sample to validate the results.

### Identification of PCR products

The amplified PCR products were identified with 1.5% agarose gel electrophoresis stained by ethidium bromide. DL2000 marker (TaKaRa) was used as molecular size marker. The amplified PCR products were purified with TaKaRa MiniBEST DNA fragment purification Kit (TaKaRa) according to the manufacturer’s protocol. When multiple bands were visualized on the gel, they were cut out and the DNA was purified with TaKaRa MiniBEST agarose gel DNA Extraction Kit (TaKaRa). The forward strands were sequenced by the fiuorescence-based dideoxy chain termination method with the forward primer (*vrrA*-F) in an automated DNA sequencer (Applied Biosystems model 3730XL). Sequence analysis was performed with the entire cloned fragment, omitting the primer sequences used to amplify the *vrrA* genes. Multiple alignment of the *vrrA* gene sequences was carried out with ClustalX. Phylogenetic analysis was performed with Mega 5.1.

### Statistical analysis

All data were expressed as mean ± s.e.m. All analysis was performed using version SPSS16.0 software (SPSS Inc., USA).

## Results

### Isolation of *B*. *cereus* strain and sequencing their *vrrA* genes

**Fig 1:**
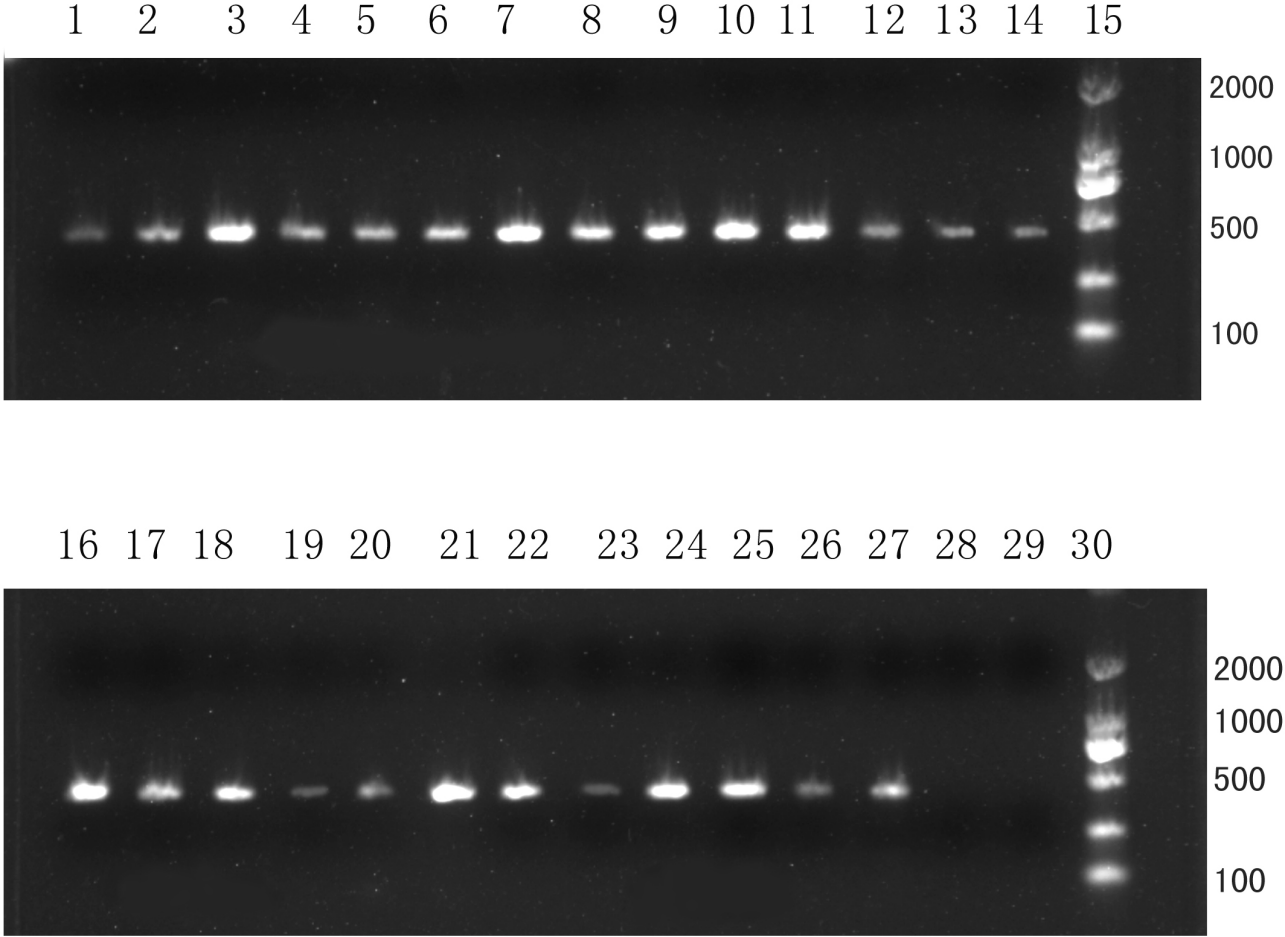
PCR amplification of the *vrrA* gene among clinical *B*. *cereus* isolates. Lane1-14: Bc1-Bc14; lane16-25: Bc16-Bc25; Lane26: *B*. *cereus* ATCC14579; Lane27: *B*. *thuringiensis* CTCC22945; Lane28: *Bacillus subtilis* ATCC9372; Lane29: ddH2O; Lane15 and Lane30: DNA marker. The predicted size of the product was approximately 430bp. A total of twenty–four *B*. *cereus* strains were confirmed by the PCR, and non-*B*. *cereus* strains did not yield a PCR product.

A total of twenty-four *B*. *cereus* strains were isolated from either anterior chamber fluid or vitreous body of patients with infective endophthalmitis. All strains were analyzed by PCR amplification for the presence of the *vrrA* gene. The anticipated length was approximately 430bp (**Fig 1**). As expected, non-*B*. *cereus* strains did not yield a PCR product. All PCR products were then subjected to sequencing. Overall, the nucleotide sequences of the twenty-four clinical *B*. *cereus* isolates had 95% to 99% identity with that of *B*. *cereus* ATCC 14579 sequence.

### Phylogenetic analysis and genotyping of the *B*. *cereus* isolates

**Fig 2:**
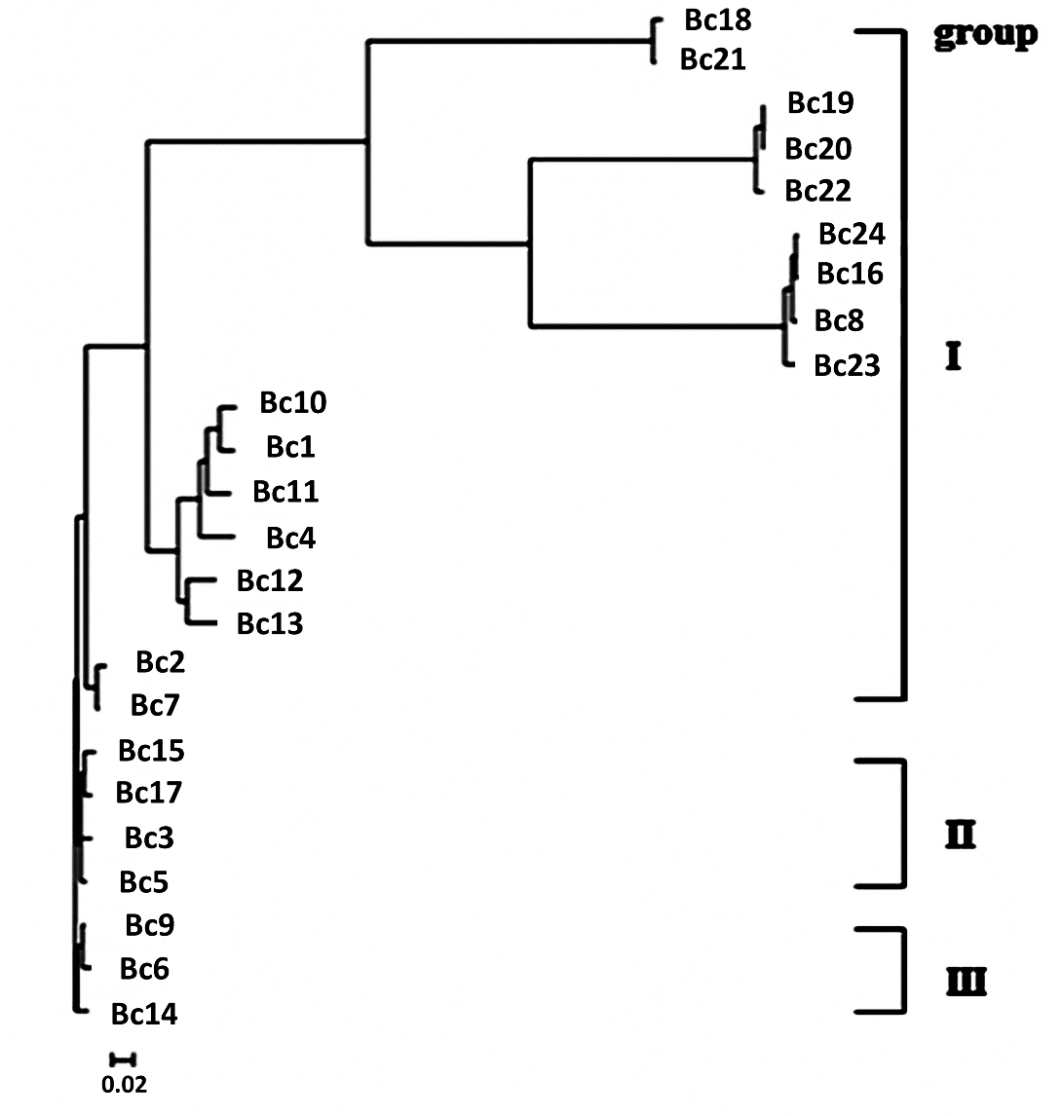
Clustering analysis of genotyping data of B. cereus clinical endophthalmitis isolates. Phylogenetic tree of *B*. *cereus* clinical endophthalmitis isolates was drawn based on *vrrA* gene sequence analyses. The figure showed that *B*. *cereus* isolats could be grouped into three genotyping (GT) groups: GTI, GTII, and GTIII. GTI was be further divided into three subgroups.

The phylogenetic tree of the twenty-four *B*. *cereus* clinical isolates was constructed based on the *vrrA* gene sequences (**Fig 2**). The result showed that the *B*. *cereus* isolates could be grouped into three genotyping (GT) groups. GTI was be further divided into three subgroups (GTIa, GTIb and GTIc). GTIa included nine *B*. *cereus* strains, GTIb had six strains, and GTIc had two strains. Four and three strains fell into GTII, and GTIII, respectively.

### Association of *vrrA*-based *B*. *cereus* genotypes with clinical manifestation and prognosis of outcome

**Fig 3:**
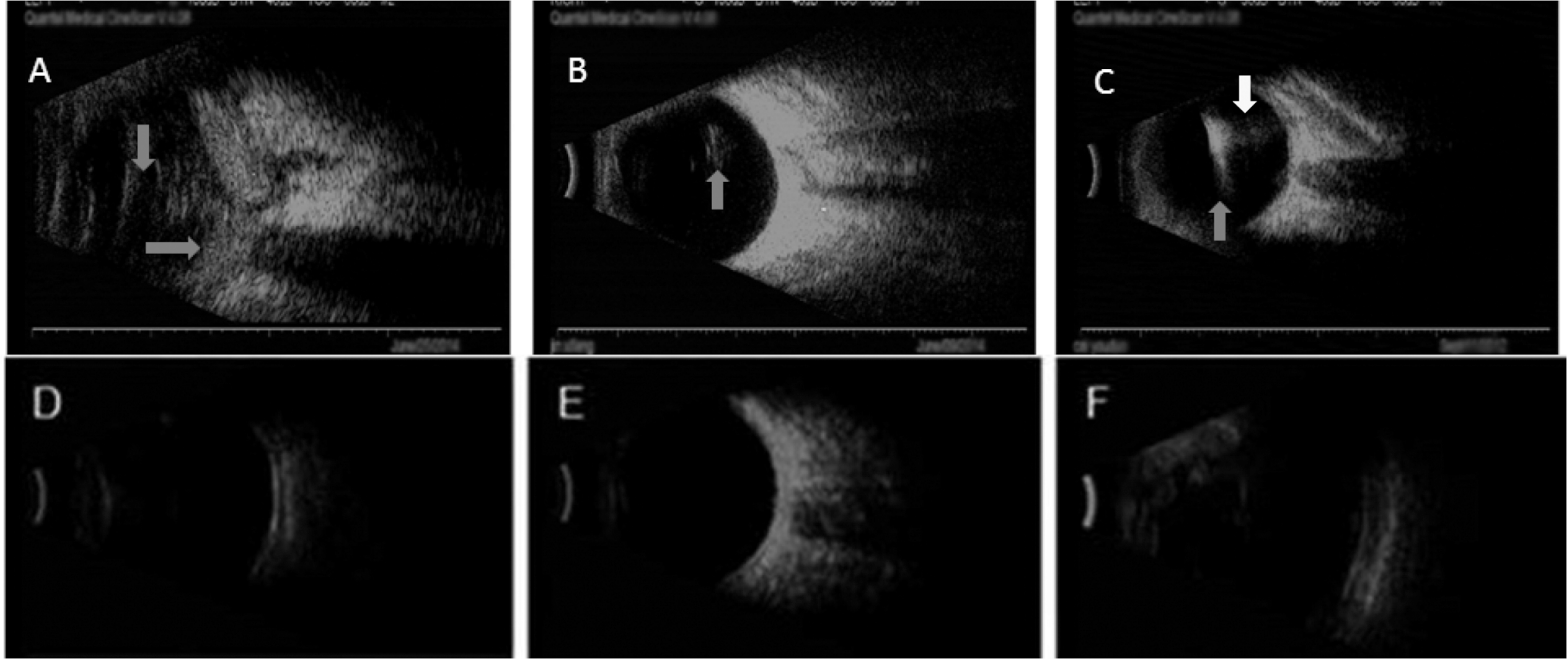
B-scan images of the patients' eyes infected with *B*. *cereus* before (A,B,C) and after (D,E,F) surgery operation. (A). Infection caused by GTI strains showed severe vitreous opacity (gay arrows). (B). Infection caused by GTII strains showed mild vitreous opacity (gray arrows). (C). Mild vitreous opacity infection caused by GTIII strains (gray arrow),high reflection and ascoustic shadow in vitreous showed foreign body (white arrow). (D). Infection caused by GTI strains after surgery. (E). Infection caused by GTII strains after surgery. (F). Infection caused by GTIII strains after surgery(the false expansion of eye ball after vitreous surgery with silicon oil tamponade).

Clinical representation, as well as prognosis of outcome of each patients, were collected. All B-scan images showed foreign bodies in the eyeballs as well as showed vitreous opacity, but the degree of opacity was different among three genotypes (**Fig 3**). Infections caused by GTI strains resulted in most severe clinical manifestation, and the prognosis of outcome were the poorest. All seventeen patients lost their vision (NLP). The clinical features of GTII and GTIII infections were mild, and the prognosis was significantly better than the of GTI infections. No statistical differences between GTII and GTIII in term of clinical score (**Table 2**). All three patients from GTIII had light perception (LP). The recovered vision acuity in patients with GTII infections was the best, in which all four patients regained functional vision (with acuity equal to or better than 2/100). Among them, the vision acuity of two cases were 2/100, one was 20/40, and another 80/100.

**Table 2:** Clinical manifestation and prognosis associated with three *vrrA*-based genotypes of *B*. *cereus*.

		GT		
variables	I(n = 17)	II(n = 4)	III(n = 3)	P value*
Onset time (h)	16.9±14.7	102±49.5	25.7±21.5	*<0*.*05*
Size of the wound(mm^2^)	4.2±2.0	5.0±1.7	3.5±1.7	*>0*.*05*
Clinical score	7.7±2.7	4.3±2.8	3.7±2.1	*<0*.*05*
*Final vision acuity*	*NLP*	*0*.*34*	*LP*	*<0*.*05*

Ps: Date are reported as mean ± SD.

* p values were calculated using Kruskal-Wallis test.

## Discussion

In this study, we isolated twenty-four *B*. *cereus* clinical isolates from endophthalmitis patients, and demonstrated that these twenty-four *B*. *cereus* isolates can be grouped into three genotypes using *vrrA* gene sequences. We found that different groups correlate with variable clinical manifestations and prognosis. Our finding suggests that the *vrrA*-based genotyping has the potential to be used for predicting clinical manifestations and prognosis of *B*. *cereus*-caused endophthalmitis.

PCR-based molecular diagnostic techniques have been used to improve the sensitivity of *B*. *cereus* detection in endophthalmitis patients recently. A study of one hundred post-cataract endophthalmitis patients in France indicated that the sensitivities of PCR was much higher than that of culture in subsequent vitrectomy samples (70% vs. 9%)[16]. In another study of eleven cases with endophthalmitis in Brazil, PCR was 91% positive and cultures were 75% positive[17]. DNA sequence can provide additional information than PCR assay and can be utilized for genotyping. Chang et. al. used *groEL* and *sodA* gene for the purpose[10]. Brumlik et al used long-range repetitive element polymorphism-PCR to differentiate various *B*. *anthracis* strains[11]. Since the sequences of these genes are evolutionary conserved, their sequences are not well suited for genotyping of closely related *B*. *cereus* isolates.

*VrrA* gene in *B*. *cereus* group strains contains a 12bp variable-number tandem repeat (VNTR) that encodes a putative 30-kDa glutamine-rich protein. Analysis of the VNTR and similar variable repeats found within *B*. *cereus* DNA thus provides a valuable tool to characterize the epidemiology. Zahner et al analyzed the fragment length polymorphisms in the *vrrA* gene among Brazilian isolates and *Bacillus thuringiensis*[13]. They found close taxonomic proximity of *B*. *cereus* and *B*. *thuringiensis*.

In this study, we explored the potential of the *vrrA* gene as a phylogenetic marker. A phylogenetic analysis of the *vrrA* sequences of twenty-four *B*. *cereus* revealed that the *B*. *cereus* fell into three major groups. GTI contained three subgroups (GTIa, GTIb and GTIc). Nine *B*. *cereus* strains fell into GTIa, six in GTIb, two in GTIc, Four in GTII, and three in GTIII. We also retrospectively analyzed the clinical manifestations and prognosis of the patients with *B*. *cereus* endophthalmitis. In Group 1, the development of disease was most aggressive and devastating and vision outcomes were poorest. Group II and III included seven strains. The clinical features were mild, and the prognosis was better than that of Group I.

It is well accepted that the preservation of vision is based on clinical suspicion and rapid intervention. Firstly, the most important component of treatment is the direct injection of antibiotics into the eye (e.g. vancomycin). Secondly, rapid intervention such as vitrectomy is imperative. The combination of removal or replacement of the IOL, total capsulectomy, vitrectomy and intravitreal antibiotics has been proved to be the most successful approach[18]. In our study, seventeen patients underwent vitrectomy and received intravitreal injection of antibiotics (vancomycin 1mg plus ceftazidime 1mg). Because of poor control of infective endophthalmitis, six patients experienced enucleation. One patient received the therapy of foreign body extraction and intravitreal injection of vancomycin.

In this study, we found that seven cases got better vision outcomes than the other seventeen. All seventeen cases with no light perception were divided into the same group, and seven cases which resulted in better prognosis were sorted into another groups. Besides, the vision acuity of GTII was better than that of GTIII. The best recovery of vision acuity was 80/100. Thus, significant relationship exists between different genotypes of *vrrA* sequences and clinical features or prognosis., suggesting that *vrrA* gene may be used as a phylogenetic marker in *B*. *cereus* genotyping as well as prediction of clinical features or prognosis. The exact mechanism underlying the contribution of *vrrA* gene to *B*. *cereus* endophthalmitis remains yet-to-be elucidated. Further study will focus on how different genotypes of *vrrA* genes contributes with the pathogenesis of *B*. *cereus* endophthalmitis.
